# Integrated THz/FSO Communications: A Review of Practical Constraints, Applications and Challenges

**DOI:** 10.3390/mi16111297

**Published:** 2025-11-19

**Authors:** Jingtian Liu, Xiongwei Yang, Yi Wei, Feng Zhao

**Affiliations:** School of Electronic Engineering, Xi’an University of Posts and Telecommunications, Xi’an 710121, China; 22110720137@m.fudan.edu.cn (X.Y.); weiyi_email@163.com (Y.W.)

**Keywords:** terahertz communication, free space optical communication, integrated convergence, reliability enhancement, switching strategies, atmospheric turbulence, long-distance transmission, atmospheric absorption, photonic-assisted

## Abstract

This paper presents a comprehensive review of integrated terahertz (THz) and free-space optical (FSO) communication systems, focusing on their potential to address the escalating demands for high-capacity, long-distance, and ultra-reliable transmission in future six-generation (6G) and space–air–ground integrated networks (SAGIN). The study systematically examines recent advancements in three critical areas: channel modeling, transmission performance, and integrated system architectures. Specifically, it analyzes THz and FSO channel characteristics, including attenuation mechanisms, turbulence effects, pointing errors, and noise sources, and compares their complementary strengths under diverse atmospheric conditions. Key findings reveal that THz communication achieves transmission rates up to several Tbps over distances of several kilometers but is constrained by molecular absorption and weather-induced attenuation, while FSO offers superior bandwidth-distance products yet suffers from turbulence-induced fading, posing significant reliability challenges. The integration of THz and FSO through adaptive switching strategies (e.g., hard and soft switching) demonstrates enhanced reliability and spectral efficiency, with experimental results showing seamless data rates exceeding Tbps in hybrid systems. However, challenges persist in transceiver hardware integration, algorithmic optimization, and dynamic resource allocation. The review concludes by identifying future research directions, including the development of unified channel models, shared architectures, and intelligent switching algorithms to achieve robust integrated communication infrastructures.

## 1. Introduction

Next-generation 6G networks targeting 2030+ applications necessitate Tbps-level throughput while establishing ultra-massive connectivity and ultra-reliable low-latency communication infrastructures. Satellite-assisted SAGIN addresses these demands through coordinated multi-layer architectures [1,2,3,4], enabling enhanced transmission capabilities via spaceborne, aerial, and terrestrial multi-access convergence. This integration paradigm ensures continuous service availability with exceptional reliability and quality-of-service (QOS) parameters, ultimately delivering seamless global communication infrastructure.

**Figure 1:**
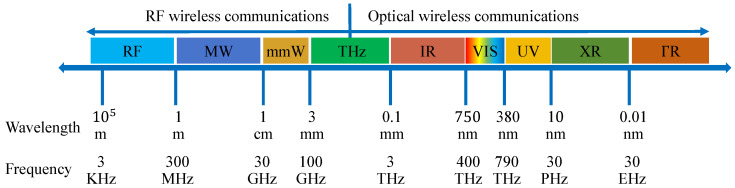
Illustration of wireless communication frequency bands. The commonly used THz band in free-space communication is between 100–300 GHz, and FSO is between 780–1550 nm; RF: radio frequency; MW: microwave; mmW: millimeter wave; IR: infrared light; VIS: visible light; UV: ultraviolet; XR: X-ray; ΓR: Γ-ray.

Contemporary inter-satellite and satellite-ground communications predominantly employ electromagnetic wave transmission, with microwave- and laser-based systems constituting the primary technical approaches. While microwave systems demonstrate proven reliability and extensive coverage, their spectrum constraints within the 3–40 GHz range prove insufficient for emerging high-speed, large-capacity transmission requirements. FSO communications, benefiting from abundant spectrum resources as illustrated in Figure 1, offers distinct advantages [5], including broad bandwidth and long-range transmission capabilities [6]. Operating in unregulated spectrum regions, this technology proves particularly effective for intersatellite and satellite-ground link establishment. However, FSO system performance degrades under atmospheric disturbances such as fog, smoke, and turbulence, with additional susceptibility to background radiation-induced link instability [7,8,9,10].

The THz band presents complementary strengths through enhanced atmospheric penetration in adverse conditions compared to FSO systems, while maintaining immunity to background radiation and turbulence effects [11,12]. Nevertheless, current technological limitations in high-power THz signal generation and amplification restrict the practical implementation of high-capacity THz communication to kilometer-scale transmission distances.

**Figure 2:**
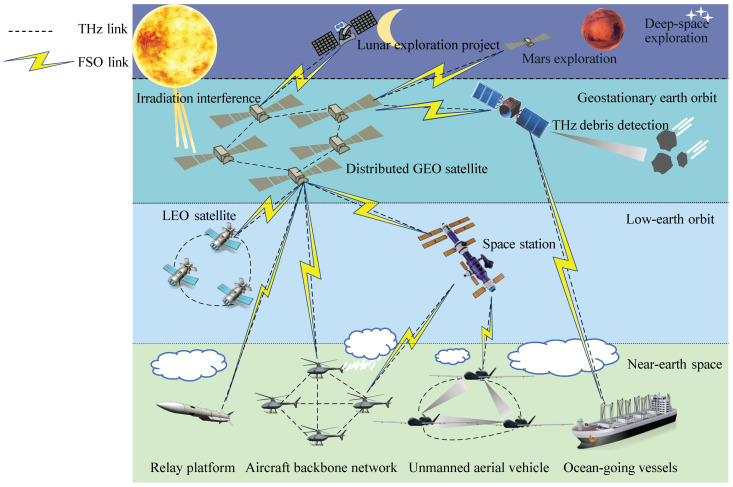
Illustration of integrated THz/FSO SAGIN applications. GEO: geostationary Earth orbit; LEO: low-Earth orbit.

The strategic integration of THz and FSO communication systems presents a transformative approach to address evolving demands in satellite communications, 6G networks, and mission-critical applications [13,14,15] as conceptually illustrated in Figure 2. This hybrid architecture capitalizes on complementary spectral and propagation characteristics to achieve four fundamental scientific advancements:

- Spectral resource optimization: Integrated THz/FSO systems transcend single-band limitations through multi-dimensional multiplexing schemes, enabling simultaneous seamless ultra high speed, long range, and high capacity transmission. The integrated spectrum utilization paradigm effectively resolves frequency congestion in conventional electromagnetic bands.
- Channel robustness enhancement: By implementing adaptive switching mechanisms between THz’s fog/smoke penetration capabilities and FSO’s directional precision, the system dynamically optimizes transmission efficiency across diverse atmospheric conditions, significantly improving link stability compared to standalone implementations.
- Device architecture innovation: The convergence necessitates breakthroughs in three core technological domains: Coherent source–receiver alignment for heterogeneous frequency operation, advanced signal processing algorithms for hybrid waveform decoding, photonic–electronic integration for efficient beamforming architectures.
- Interdisciplinary synergy: System integration drives theoretical advancements across electromagnetic theory, atmospheric photonic, and quantum-limited detection, establishing novel frameworks for next-generation communication physical layer design.

This study comprehensively explores THz and FSO communication systems for high-capacity, long-distance transmission, systematically assesses their integration potential, and outlines future research directions. In Section 2, we rigorously characterize wave propagation behavior under different channel conditions, quantifying key impairment mechanisms such as molecular absorption, aerosol-induced scattering, meteorological interference, and turbulence-induced signal distortion. Then, Section 3, we critically examine the latest advancements and fundamental limitations of both transmission technologies in achieving ultra-high throughput over extended operating ranges. In Section 4, we first systematically review the development history and current status of hybrid transmission systems. Based on these analyses, we then systematically discuss integrated THz/FSO system architectures, with particular focus on adaptive switching techniques. These methods optimize spectral efficiency and connectivity robustness through intelligent transmission switching algorithms. In Section 5, we summarize the research challenges and future directions for advancing next-generation space-terrestrial integrated communication infrastructure. Finally, in Section 6, we provide a detailed summary.

## 2. Current Progress in THz and FSO Channel Model

**Figure 3:**
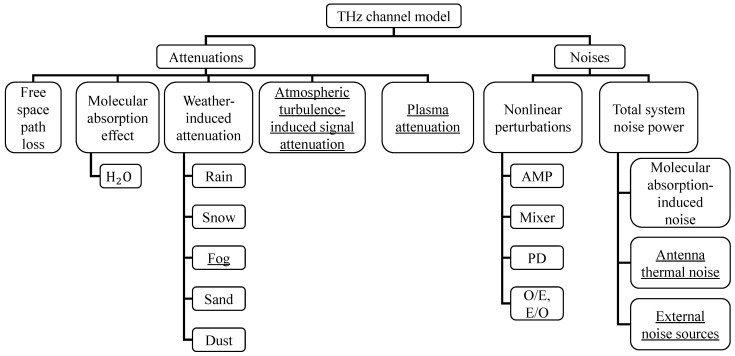
Classification of THz channel impact based on attenuation and noise. Underline: Effects that can be ignored in this paper’s model.

**Figure 4:**
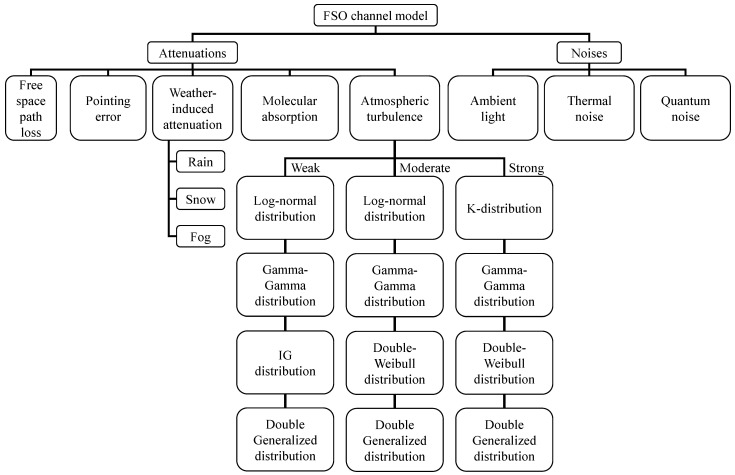
Classification of FSO channel impact based on attenuation and noise.

Many effects influence the transmission performance of THz and FSO transmission. To help readers better understand the impact of these effects on transmission, this chapter details the channel models for both. Figure 3 and Figure 4 list the attenuation and noise effects primarily considered in previous studies, and underline effects that are negligible in the SAGIN system.

### 2.1. THz Channel Model

THz wave propagation is predominantly governed by four attenuation mechanisms: free space path loss (FSPL), molecular absorption, weather-induced attenuation, and nonlinear distortions from devices/channels as illustrated in Figure 3. In terrestrial scenarios, dense obstacles and reflectors generate significant multipath propagation. Conversely, in non-terrestrial environments (e.g., aerial/space links), multipath effects are minimal, often reducing to a single dominant line-of-sight (LOS) path between transmitter and receiver [16]. Consequently, we adopt the LOS path loss model for channel gain calculation.

Further, the extended beam coverage area at THz frequencies substantially exceeds the physical aperture of receiving antennas. This configuration renders pointing-error-induced fading negligible under typical alignment conditions. If required, such effects can be incorporated via the FSO alignment error model detailed in Section 2.2.3.

#### 2.1.1. Free Space Path Loss

In the THz band, the FSPL is calculated according to the Friis transmission equation [17] to account for reflection or diffraction as follows:

(1) $$L_{p}={\left(\frac{4\pi fd}{c}\right)}^{2},$$

where *d* is the distance between transmitter and receiver (m), *f* is the frequency (Hz), *c* is the velocity of light (m/s).

#### 2.1.2. Molecular Absorption Effect

Atmospheric absorption of THz waves arises from quantum-level energy transitions during photon-molecule collisions involving species such as $O_{2},N_{2}$, and $H_{2}O$. Resonant absorption occurs when the incident photon energy precisely matches the energy gap between molecular quantum states, yielding a substantial absorption cross-section. Among these molecules, water vapor dominates atmospheric attenuation due to its broad resonant lines in the millimeter-wave and THz spectra. The aggregate molecular absorption gain across these frequency bands can be quantified using the Beer–Lambert law [17]:

(2) $$G_{a}=e^{-\kappa_{a}\left(f\right)d},$$

where $\kappa_{a}\left(f\right)$ denotes the molecular absorption coefficient, defined as the sum of absorption cross-sections per unit volume within the medium. This coefficient governs attenuation in the 100–450 propagation window, where distinct absorption peaks emerge at approximately 119, 183, 325 and 380 GHz, with the dominant peak occurring at 380 GHz [18,19].

#### 2.1.3. Weather Effect

Tropospheric precipitation (including rain, fog, snow, and dust) induces wavelength-dependent signal attenuation. When particle radii are comparable to the THz wavelength, the Mie absorption-scattering model characterizes the cross section; otherwise, the Rayleigh model applies. Since raindrop radii (1 to 10 mm) are comparable to D-band THz wavelengths (∼3 mm), whereas fog droplets (20 μm to 2 mm) are significantly smaller, rain-induced Mie scattering causes severe signal degradation and heightened attenuation. The specific attenuation $\gamma_{R}$ (dB/km) in the ITU-R recommended model is calculated from the rain rate R (mm/h), as shown in Table 1 via the power-law relationship [20]:

(3) $$\gamma_{R}=kR^{\alpha },$$

where the coefficients k and α are frequency-dependent (see Table 5 in [20]), with typical values of 1.6286 and 0.6262 at 300 GHz. As an empirical formulation grounded in extensive experimental data, this equation demonstrates strong consistency with analytical droplet size distribution (DSD) models such as the MP, Weibull, and Gamma DSD models [21]. For example, at a rainfall rate of 20 mm/h, the rain attenuation reaches 10.6 dB/km.

Rayleigh scattering from fog particles minimally impacts THz wave propagation [4] unless the fog is extremely dense, characterized by very low visibility, where attenuation can exceed 100 dB/km [22]. Snow and sleet represent additional attenuation sources, primarily due to the water vapor they contain. However, compared to rain and fog, accurately predicting the attenuation caused by snow or sleet presents greater challenges due to the complex and variable size distribution and geometries of snowflakes. Measurements indicate that within the 300 GHz band and at equivalent precipitation rates (e.g., 20 mm/h), sleet induces higher attenuation than snow, which, in turn, exceeds that caused by rain [23,24]. Under these conditions, dry snow attenuation is approximately 15 dB/km. Furthermore, dust and sand also contribute to THz signal loss, with the attenuation magnitude influenced by the particle density within aerosols and the size of the sand grains [25,26].

#### 2.1.4. Nonlinear Perturbations

In high-speed communication systems, nonlinear distortions manifest due to hardware imperfections in components such as amplifiers (AMP), mixers, photodetectors (PD), and optoelectronic converters (O/E, E/O) [27,28,29]. For THz transceivers specifically, these imperfections primarily consist of in-phase/quadrature imbalance (IQI), phase noise (PHN), and amplifier nonlinearities, which collectively degrade performance in high-data-rate operation [30,31]. The aggregate distortion η can be statistically modeled as a circularly symmetric complex Gaussian process (CN) [32]:

(4) $$\eta ∼\mathrm{CN}(0,\kappa^{2}P),$$

where κ denotes the aggregate impairment coefficient (typically 0 to 0.3), this coefficient corresponds directly to the error vector magnitude (EVM)—a metric that quantifies waveform distortion and is experimentally measurable [33]. *P* represents the average transmitted signal power.

#### 2.1.5. Other Discussions

In addition to the effects mentioned above, there are numerous other physical effects that can attenuate or add noise to THz transmissions. As established in [34], atmospheric turbulence-induced signal attenuation exhibits a frequency dependence proportional to $f^{\frac{7}{6}}$. However, for THz links under typical non-extreme conditions, this attenuation remains negligible. Plasma attenuation is equally insignificant, measuring below 10^−3^ dB/km and thus disregarded in THz systems.

Molecular absorption within the propagation medium introduces dual effects: it governs channel attenuation while simultaneously generating molecular absorption noise [35]. This noise originates from thermal re-emission of absorbed energy, where molecular vibrations excited by incident radiation produce electromagnetic waves at resonant frequencies. Beyond molecular noise, the antenna noise temperature incorporates contributions from thermal noise in antenna components, electromagnetic interference from integrated nanodevices, and system-level noise sources. Consequently, total receiver noise power comprises three components:

- Molecular absorption-induced noise;
- Antenna thermal noise;
- External noise sources.

Since nanomaterial-enabled receivers achieve ultralow intrinsic noise floors [36], our model focuses exclusively on channel-generated molecular absorption noise—consistent with foundational THz channel modeling frameworks [17]:

(5) $$P_{n}\left(f,d\right)=k_{B}\int_{B}T_{0}\left(1-G_{a}\left(f,d\right)\right)df,$$

where $T_{0}$ is the reference temperature, $k_{B}$ is the Boltzmann constant, and *B* is the bandwidth.

#### 2.1.6. Channel Model

According to the above effects, the received signal is conventionally modeled based on Equation (5) in [32]:

(6) $$y=\sqrt{\frac{G_{a}G_{t}G_{r}}{L_{p}10^{\gamma_{R}d/10}}}\left(x+\eta \right)+\nu ,$$

where ν is modeled as a complex zero-mean additive white Gaussian process with variance equals $P_{n}$, $G_{t}$ and $G_{r}$ respectively represent the antenna orientation dependent transmission and reception gain.

### 2.2. FSO Channel Model

FSO communications experience six primary impairments, as illustrated in Figure 4: path loss, atmospheric turbulence, molecular absorption, weather-induced attenuation, pointing error, and noises from ambient light and thermal noise. For long-range links, path loss follows a deterministic model (Equation (1)) governed by beam divergence, for short-range links, path loss is incorporated within the pointing error component as geometric spread. These composite impairments cause signal attenuation that directly degrades the signal-to-noise ratio (SNR) at the receiver.

#### 2.2.1. Atmospheric Turbulence

Atmospheric turbulence originates from temperature and pressure gradients that create refractive index inhomogeneities, these inhomogeneities form turbulent eddies that deflect optical beams through three distinct mechanisms based on eddy-to-beam size ratio [37,38]:

- Beam Wander (Eddies > Beam Width): Large eddies displace the entire beam centroid randomly, causing pointing errors that may misalign the beam from the receiver aperture.
- Scintillation (Eddies ≅ Beam Width): Eddy-induced focusing/defocusing creates random irradiance fluctuations at the receiver, degrading SNR through deep fading.
- Beam Spreading (Eddies < Beam Width): Small-scale diffraction and scattering reduce received power density while distorting the wavefront.

Scintillation, manifested as intensity fluctuations, dominates fading effects in typical FSO channels [39]. Statistical models for scintillation-induced fading include [40,41]:

- Log-normal distribution: Weak-to-moderate turbulence [41].
- Inverse Gaussian (IG) distribution: Weak turbulence [42].
- Double Weibull distribution: Moderate-to-strong turbulence [43].
- Double Generalized distribution: Weak-to-strong turbulence [44].
- K-distribution: Strong turbulence [45].
- Gamma-Gamma (GG) distribution: Weak-to-strong turbulence [46].

The GG distribution, which is in close agreement with measurements under a variety of turbulence conditions, is the product of two random variables of large scale and small scale eddies. The probability density function (PDF) of the GG distribution with Rytov variance $\sigma_{R}^{2}$ [47] for the intensity $h_{a}$ is given as [46]:

(7) $$f\left(h_{a}\right)=\frac{2{\left(\alpha \beta \right)}^{\frac{\alpha +\beta }{2}}h_{a}^{\frac{\alpha +\beta }{2}-1}}{\Gamma (\alpha )\Gamma (\beta )}K_{\alpha -\beta }\left(2\sqrt{\alpha \beta h_{a}}\right),h_{a}>0$$

and the parameters α and β are given as:

(8) $$\alpha ={\left[\exp\left(\frac{0.49\sigma_{R}^{2}}{{\left(1+1.11\sigma_{R}^{\frac{12}{5}}\right)}^{\frac{7}{6}}}\right)-1\right]}^{-1},$$

(9) $$\beta ={\left[\exp\left(\frac{0.51\sigma_{R}^{2}}{{\left(1+0.69\sigma_{R}^{\frac{12}{5}}\right)}^{\frac{5}{6}}}\right)-1\right]}^{-1},$$

where α and β are the effective number of the small scale and large scale eddies of the scattering process, and $K_{a}\left(.\right)$ is the modified Bessel function of order *a*.

The fading process in atmospheric turbulence channels exhibits a coherence time of 10^−3^ to 10^−2^ s, orders of magnitude longer than typical symbol durations (10^−9^ s) [48,49]. Consequently, the channel gain $h_{a}$ remains effectively constant during individual transmissions. This slow variation renders interleaving techniques impractical for averaging over fading states. Such channels exhibit block-fading characteristics (slow-fading/non-ergodic), this fading can be efficiently modeled via exponentially correlated processes using first-order stochastic differential equations [50].

#### 2.2.2. Weather Effects

The atmospheric attenuation coefficient represents the aggregate loss resulting from the absorption and scattering coefficients associated with both atmospheric molecular constituents and aerosols. At the wavelength of 1550 nm, molecular absorption is approximately 0.01 dB/km and can therefore be neglected. The primary contributor to atmospheric attenuation is particle scattering, which occurs across various hydrometeors such as haze particles, fog droplets, snow, rain, and hail. Among these, Mie scattering by fog particles induces significant attenuation, as characterized by the following empirical relationship [51]:

(10) $$\alpha_{\mathrm{fog}}\left(V\right)=\frac{13}{V}{\left(\frac{\lambda }{0.55}\right)}^{-q(V)}\left[\mathrm{dB}/\mathrm{km}\right],$$

(11) $$q\left(V\right)=\left\{\begin{array}{cc} 1.6 & \;\;\;\;\;\mathrm{for}\;V>50\;\mathrm{km}\\ 1.3 & \;\;\;\;\;\mathrm{for}\;6<V<50\;\mathrm{km}\\ 0.16V+0.34 & \;\;\;\;\;\mathrm{for}\;1<V<6\;\mathrm{km}\\ V-0.5 & \;\;\;\;\;\mathrm{for}\;0.5<V<1\;\mathrm{km}\\ 0 & \;\;\;\;\;\mathrm{for}\;V<0.5\;\mathrm{km}. \end{array}\right.$$

where $\alpha_{\mathrm{fog}}$ denotes the attenuation coefficient due to fog particle scattering in dB/km; λ is the wavelength in micrometers (μm); q(V) represents a fog particle size distribution parameter dependent on meteorological visibility; and *V* signifies the meteorological visibility range, which is defined as the distance over which atmospheric transmittance at 550 nm decreases to a reference threshold value of ϵ=0.05 [51]. (Note: Increased fog concentration is positively correlated with decreased turbulence intensity [52].)

Beyond fog, rain constitutes another significant source of signal attenuation in FSO communication systems. Because raindrop radii typically exceed the wavelengths used in FSO, Mie scattering governs the attenuation process. Compared to fog, however, attenuation due to rain is generally less severe. The rain-induced specific attenuation, dependent on the rainfall rate *R* (as shown in Table 1) is given by [53]:

(12) $$\alpha_{\mathrm{rain}}=kR^{\alpha }\;\left[\mathrm{dB}/\mathrm{km}\right],$$

where the coefficients *k* and α are characteristic of the rain type and climate; typical values, for example, are *k* = 1.076 and α = 0.67 [53].

Attenuation caused by snow exhibits considerable variability, influenced by characteristics such as snowflake size morphology and snowfall intensity. Notably, large snowflakes (exceeding 20 mm) can potentially induce severe attenuation or even completely block an optical path, contingent upon the beam width [54].

#### 2.2.3. Pointing Error

In line-of-sight FSO communication links, random building vibrations induced by wind loads or thermal expansion can introduce pointing errors, leading to signal fading at the receiver [55]. For a Gaussian beam, the pointing error loss $h_{p}$ due to misalignment can be modeled by the following distribution under the assumption that elevation and horizontal sway displacements follow independent and identical Gaussian distributions [39]:

(13) $$f_{h_{p}}\left(h_{p}\right)=\frac{\gamma^{2}}{A_{0}^{\gamma^{2}}}h_{p}^{\gamma^{2}-1},\;0\leq h_{p}\leq A_{0}$$

where γ denotes the ratio of the equivalent beam radius at the receiver to the standard deviation of the pointing error displacement (jitter) at the receiver. The parameter $A_{0}$ represents the fraction of collected power at zero distance (d=0), accounting for inherent beam spreading effects within the model.

Beyond the macroscopic building sway discussed above, pointing errors in FSO systems are critically influenced by two additional factors: the intrinsic properties of the laser beam and the limitations of the beam steering apparatus itself.

Firstly, beam divergence is a fundamental source of geometric loss. Even with perfect alignment, the natural spreading of the Gaussian beam over distance means that only a fraction of the transmitted power is collected by the finite-sized receiver aperture, which is the parameter $A_{0}$ in Equation (13). This effect is compounded by any lateral displacement, leading to a rapid decrease in received power. Therefore, the pointing error model must be considered in conjunction with the geometric path loss inherent to divergent beam propagation.

Secondly, the precision of the steering devices—such as fast steering mirrors, galvanometers, and spatial light modulators (SLMs)—introduces micro-scale errors. The limited resolution, step accuracy, and hysteresis of these mechanisms can cause deviations that are often more deterministic and persistent than random jitter. For instance, the finite pixel size of an SLM used for wavefront correction or beam steering imposes a quantization limit on the achievable pointing angle. These device-level inaccuracies can indeed be a dominant error source in high-precision, long-distance FSO links, as they directly limit the system’s ability to achieve and maintain optimal alignment. Addressing these combined challenges necessitates advanced acquisition, tracking, and pointing (ATP) systems, sophisticated control algorithms are required to mitigate the compound effects of random platform vibrations, inherent beam divergence, and the finite precision of steering hardware.

#### 2.2.4. Noise Sources

The dominant noise sources in FSO receivers include ambient light noise, quantum noise, and thermal noise. Ambient light noise manifests as shot noise induced by solar and atmospheric radiation, with its variance expressed as:

(14) $$\sigma_{\mathrm{Am}}^{2}=2qBR\left(I_{\mathrm{sky}}+I_{\mathrm{sun}}\right)$$

where *B* denotes the electrical bandwidth; R is the photodetector responsivity; *q* represents the electron charge; and $I_{\mathrm{sky}}$ and $I_{\mathrm{sun}}$ denote sky and solar irradiance (power per unit area), respectively.

Quantum (shot) noise arises from the stochastic nature of photon detection and yields a variance of:

(15) $$\sigma_{\mathrm{Qtm}}^{2}=2qBRI$$

Thermal noise originates from electron fluctuations in the receiver circuitry, characterized by:

(16) $$\sigma_{\mathrm{Th}}^{2}=\frac{4k_{B}T_{e}B}{R_{L}}$$

where $R_{L}$ is the equivalent load resistance, $T_{e}$ is the effective temperature, and $k_{B}$ is Boltzmann’s constant. When ambient light noise and thermal noise components dominate over the received signal, the aggregate noise can be accurately modeled as additive white Gaussian noise (AWGN) that is statistically independent of the transmitted signal [56,57]. The total noise variance at the receiver is then:

(17) $$\sigma_{n}^{2}=\sigma_{\mathrm{Qtm}}^{2}+\sigma_{\mathrm{Am}}^{2}+\sigma_{\mathrm{Th}}^{2}$$

#### 2.2.5. Channel Model

According to the above effects, the received signal is conventionally modeled as:

(18) $$y=h_{l}h_{p}h_{a}Rx+n,$$

where *x* is the transmitted intensity; $h_{l}=10^{\frac{-\alpha d}{10}}$ is the atmospheric loss based on actual weather conditions by Equations (10) and (12); $h_{p}$ and $h_{a}$ are random variables (RVs) following the distribution in Equations (7) and (13), respectively; and *n* is a signal-independent AWGN with variance $\sigma_{n}^{2}$.

### 2.3. Discussions and Conclusions

This chapter has systematically examined the channel models for THz and FSO communications, elucidating their distinct attenuation mechanisms, noise sources, and performance under varied atmospheric conditions. As summarized in Table 2, a comparative analysis from a modeling perspective reveals their complementary characteristics: THz communication offers extremely high spectral resources enabling Tbps-level rates but is constrained by deterministic attenuation from molecular absorption and weather effects, posing challenges for transmitter amplifiers over long distances. In contrast, FSO channels provide abundant spectrum and high capacity with virtual immunity to molecular absorption, yet they suffer from random fading induced by atmospheric turbulence and pointing errors, which severely disrupts link reliability.

Importantly, the analysis demonstrates that FSO and THz systems exhibit complementary attenuation profiles under different weather conditions. For instance, THz waves offer better penetration in foggy conditions where FSO links degrade, while FSO excels in clear weather with minimal turbulence. This inherent complementarity, as conceptually illustrated in Table 2, forms the foundational rationale for integrated THz/FSO systems. Through dynamic cross-layer resource allocation, such integrated architectures can achieve robust resilience across diverse propagation environments by fully leveraging the ultra-wideband potential of both technologies.

However, these distinct channel characteristics also unveil significant hurdles. The deterministic but high path loss of THz and the stochastic, turbulence-induced fading of FSO present complex challenges for achieving seamless, high-capacity, and ultra-reliable transmission in integrated systems. These fundamental limitations, rooted in the physical layer properties analyzed in this chapter, necessitate innovative solutions that transcend simple channel modeling. Consequently, the following chapter will dedicate a comprehensive analysis to the practical constraints, applications, and future research directions for THz/FSO communications, as well as integrated systems, building upon the foundational insights established in this review of channel characteristics.

## 3. Current Progress in THz and FSO High-Capacity Long-Distance Communications

### 3.1. High-Capacity Communications

Both THz and FSO communications have the potential for ultra-large-capacity data transmission on the order of Tbps, as evidenced by recent representative research progress summarized in Table 3. Consequently, THz and FSO are recognized as key enabling technologies for future 6G large-capacity, ultra-high-speed communications [58].

Extensive research has focused on optimizing THz communication links to achieve high capacity [59,61,62,63,64]. Critical enabling techniques include high-order modulation [60,65] and probabilistic constellation shaping (PCS) [77], which adaptively adjust transmission rates and enhance system capacity under limited SNR constraints, achieving net rates of hundreds of gigabits per second (Gbps). Furthermore, multiplexing schemes, implemented using sophisticated nonlinear compensation algorithms, enable the simultaneous transmission of multiple PCS signals within a single physical channel, thereby substantially increasing THz system capacity [66,67,78]. Significant achievements leveraging multiplexing include Zhang et al.’s demonstration of a 1.034 Tbps rate over 100 m using combined frequency, polarization, and spatial multiplexing [66], and Ding et al.’s use of wavelength division multiplexing (WDM) attaining 6.4 Tbps over 54 m [67].

Similarly, FSO systems employ analogous approaches—link optimization [69,71,74,79,80], high-order modulation [72,73], multiplexing [68,74,75,76,81], and PCS [70,73]—to enhance communication capacity. For instance, El-Mottaleb et al. achieved 240 Gbps over 1.41 using dual polarization and linear polarization modes [69]. Guiomar et al. demonstrated a 400 Gbps rate over 55 applying adaptive PCS to dynamically tune the symbol probability distribution, thereby improving FSO system throughput [70]. Substantial advances have also occurred in FSO multiplexing. Shao et al. replaced conventional semiconductor laser arrays with an emerging microcavity soliton optical frequency comb as a multi-carrier light source for WDM. This provided a more compact solution, enabling 1.02 Tbps over 1 km [74]. Additionally, Wang et al. reported transmitting 7.68 Tbps of polarization-division-multiplexed (PDM) orthogonal frequency-division multiplexed (OFDM) 1024-quadrature amplitude modulation (QAM) signals over 104 m using a 96-channel WDM-FSO system based on 1-bit Delta–Sigma modulation [75].

### 3.2. Long-Distance Communications

Table 4 summarizes recent representative long-distance transmission experiments conducted using THz and FSO communications. Although both technologies face challenges in achieving long-distance transmission, they exhibit distinct advantages. THz communication is significantly constrained by high attenuation. Consequently, given the current lack of high-power signal sources and amplifiers, considerable research effort has focused on transmission link optimization [82,83,84,85,86,87,88,89,90,91,92]. For example, Wei et al. successfully achieved a 4.6 km wireless transmission link in the D-band utilizing THz lens antennas, low-noise amplifiers, high-sensitivity receivers, and advanced digital signal processing (DSP) algorithms, achieving a transmission rate of 200 Gbps [87]. Similarly, Juan et al. developed a 140 GHz wireless communication system. By cascading solid-state power amplifiers with vacuum electron amplifiers, they attained a transmitter output power approaching 2 W, enabling a transmission rate of 500 Mbps over a 27 km sea surface link [88]. Yang et al. accomplished 30 km wireless transmission of 4 Gbps QPSK signals in the 125 GHz band using photon-assisted technology, advanced electronic devices, and DSP [89]. Notably, the vacuum environment characteristic of inter-satellite links drastically reduces THz wave propagation loss, making this technology a highly suitable candidate for such applications [93].

Conversely, long-distance FSO communication suffers from extreme sensitivity to adverse weather conditions and atmospheric turbulence, where dense fog and snow can induce severe attenuation or complete signal interruption. To extend the operational range of FSO systems, the development of sophisticated beam control [94,95,96,97] and turbulence compensation techniques [98,99] constitutes critical research areas for overcoming current limitations. For instance, Balasiano et al. employed an optical phased array to coherently synthesize 32 lasers. Real-time phase correction maintained beam drift within five times the diffraction-limited diameter on a 10 km link, achieving a data rate of 100 Gbps [94]. Technical optimization approaches for long-distance FSO communication also vary depending on the communication environment. In dynamic platform scenarios, Yu et al. established a robust 2.5 Gbps transmission link between airships 12 km apart. This system utilized GPS/INS laser orientation combined with an adaptive avalanche photodiode channel compensation mechanism designed to tolerate turbulence encountered between altitudes of 200 m and 1000 m [97]. For satellite communications, Horst et al. leveraged advanced adaptive optics to realize a highly reliable FSO link over 53.42 km, achieving a remarkably low link failure rate and a single-channel capacity of 1.008 Tbps [99].

**Table 4:** Representative progress in THz and FSO long-distance communication research.

Wavelength	Techniques	Capacity	Distance	Reference
THz	High-gain parabolic antenna, front-end hardware optimization, modulation error correction and environmental adaptability design	44 Gbps	1 km	[82]
	Coherent reception and combining of 8-channel FDM	160 Gbps	1.4 km	[83]
	8PSK and uses on-chip power synthesizer to optimize the link	8 Gbps	2.03 km	[84]
	Cassegrain antenna, efficient modulation, dynamic environmental adaptation	8.387 Gbps	2.92 km	[85]
	Dual-carrier superheterodyne wireless link optimization and FDMA	41.6 Gbps	3 km	[86]
	Leverage polarization multiplexing, MIMO and link optimization	200 Gbps	4.6 km	[87]
	Solid-state amplifiers and vacuum electronic amplifiers cascaded to optimize links and use variable transmission rates	0.5 Gbps	27 km	[88]
	Photon-assisted technology, cascaded electrical amplifiers, advanced DSP	2 Gbps	30.2 km	[89]
FSO	Coherent beam synthesis, combined with optical phased array	100 Gbps	10 km	[94]
	Coherent optical transmission and reception	111.8 Gbps	10.3 km	[98]
	Using fine steering mirrors to compensate for beam fluctuations combined with 40-channel DWDM	1.72 Tbps	10.45 m	[95]
	Closed-loop control of the beam combined with 54-channel DWDM	13.16 Tbps	10.45 km	[96]
	GPS/INS real-time precision pointing, coarse-precision combined tracking, adaptive turbulence compensation	2.5 Gbps	12 km	[97]
	Large aperture Cassegrain antenna, multimode fiber, FPGA real-time processing	2.5 Gbps	29 km	[100]
	High-order modulation and adaptive optical system to correct wavefront distortion caused by atmospheric turbulence	1.008 Tbps	53.42 km	[99]

## 4. Current Progress in Integrated THz/FSO Communications

### 4.1. Integrated THz/FSO Communications Capabilities

The signal fading induced by pointing errors, atmospheric turbulence, smoke, and fog significantly limits the performance of FSO links [25]. To address this challenge, high-reliability RF links are often deployed as backup solutions to maintain connectivity. However, RF links typically offer data rates limited to hundreds of Mbps, creating a bottleneck within hybrid RF/FSO systems and constraining their effectiveness and broader applicability. Integrated THz/FSO communication emerges as a superior alternative [13,14,15]. As summarized in Table 2, both THz and FSO technologies support Gbps-level data rates, enabling high-speed, seamless transmission. Furthermore, their complementary transmission characteristics enhance resilience under complex weather conditions. The high directivity inherent to both technologies also drastically reduces risks associated with signal interception and interference. The integration of THz and FSO systems combines the advantages of optical and wireless communication, significantly enhancing transmission capacity, reliability, and adaptability. Table 5 summarizes recent representative advancements in integrated THz/FSO fusion systems.

Earlier research investigated hybrid RF/FSO architectures. In 2015, Mai et al. introduced a parallel RF/FSO fusion concept [100]. Employing a Markov chain analytical framework, they theoretically demonstrated that a rate-adaptive RF/FSO system offers superior throughput and reliability compared to single-link FSO or RF systems, particularly when accounting for weather factors. Their work quantified the specific impact of weather conditions on performance and validated the reliability of the integrated transmission approach. Subsequently, in 2016, Zhang et al. experimentally validated the reliability gains of an integrated RF/FSO system under simulated fog, rain, and turbulence conditions using an adaptive diversity signal merging algorithm [101]. To further explore potential transmission gains, they proposed a novel signal coordinated signal mapping and combining (CMC) technique [102]. This method senses signal quality per data block to distribute the load accordingly, interleaving and repeating data in the frequency and time domains at the transmitter. At the receiver, adaptive weighting combines the repeated blocks, effectively mitigating system degradation caused by burst interference and power attenuation during severe weather, thereby enhancing robustness. However, these initial schemes primarily provided conceptual demonstrations of the parallel fusion architecture, without fully leveraging the substantial bandwidth resources and complementary characteristics inherent in FSO and THz technologies.

**Table 5:** Representative progress in integrated fusion communication research.

Wavelength	Techniques	Capacity	Distance	Reference
RF/FSO	Multi-dimensional multiplexing, Kramers–Kronig reception	1.196 Tbps	800 m	[103]
RF/FSO	Real-time neural network prediction and real-time adaptive diversity path selection based on FPGA	10 Gbps	500 m	[104]
RF/FSO	ARQ mechanism and adaptive signal combining algorithm	20 Gbps	4 m	[105]
RF/FSO	Adaptive diversity combination signal merging algorithm	4 Gbps	1.83 m	[101]
RF/FSO	CMC mechanism	2 Gbps	3 m	[102]
RF/FSO	High-order QAM combined with adaptive PCS rate control	200 Gbps	3 m	[106]
THz/FSO	Intelligent switching, DNN decision model	11.6 Gbps	20 m	[107]
THz/FSO	The integrated architecture of PCS, THz lens, low noise amplifier and shared transmitter	1 Tbps	54 m	[14]

Research advanced significantly in 2019 when Wang et al., building on the RF/FSO parallel architecture, integrated wavelength-division multiplexing, frequency-division multiplexing, polarization multiplexing, and Kramers–Kronig receiver techniques [103]. Their system achieved an aggregate transmission capacity of 1.196 Tbits/s over an 800 m link, offering the first experimental proof that flexible resource allocation within a fused architecture can yield substantial capacity gains and improved cost-effectiveness. Recognizing the complementary advantages under diverse channel conditions, Brandão et al. implemented adaptive rate control on a 200 Gbit/s RF/FSO link using probabilistic shaping control strategies [106]. This work demonstrated the complementary benefits of the fused link across different channel states.

Addressing real-time adaptability, Song et al. (2022) demonstrated adaptive real-time diversity path selection under varying weather conditions within an RF/FSO fusion system [104]. Leveraging a time-attention-enhanced gated recurrent unit (GRU) neural network implemented on an FPGA platform, they developed a prediction-based mechanism to select the optimal path, confirming the feasibility of adaptive rate control and path selection for similar THz/FSO fusion systems. In 2023, Liu et al. combined an automatic repeat request (ARQ) mechanism with an adaptive signal merging algorithm [105]. Their experiments showed that an RF/FSO fusion architecture could significantly enhance link reliability under challenging channel conditions. In 2024, Chong et al. first deployed a deep neural network (DNN) decision model in a THz/FSO system [107]. The trained neural network model outputs switching decisions in real time, enabling early switching when the channel changes, thus achieving zero-outage communication without switching latency.

Seeking cost reduction, Wang et al. (2024) proposed an innovative THz/FSO fusion scheme based on a shared light source and shared optical path mechanism [14]. This integrated system featured a shared transmitter architecture enabling 1 Tbit/s signal transmission over a 50 m wireless link. By deeply integrating THz and FSO designs, the team maximized their complementary strengths in high-capacity transmission, significantly boosting communication capacity at a lower cost. This shared transmitter approach not only meets ultra-high data rate requirements but also offers novel design paradigms for future communication systems.

### 4.2. Integrated Communication Systems Switching Strategies

**Figure 5:**
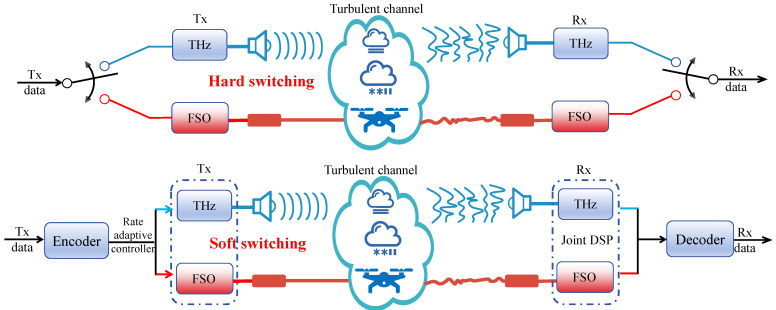
Schematic diagram of the hard/soft switching principle of the integrated fusion system.

To address the constraints imposed by dynamic channel impairments on transmission performance in FSO communications, researchers have increasingly explored multi-band fusion communication in recent years. This has led to an evolution of technical approaches, from early RF/FSO hybrid systems toward the integration of THz/FSO communication systems. By leveraging frequency band complementarity and intelligent coordination mechanisms, these systems synergistically enhance transmission reliability and spectral efficiency—particularly under complex meteorological conditions (e.g., rain, fog, atmospheric turbulence) and in long-distance scenarios. Current research primarily focuses on two dominant technical paradigms: intelligent hard-switching mechanisms and adaptive soft-switching strategies, as illustrated in Figure 5.

#### 4.2.1. Intelligent Hard Switching Mechanism

In a hard-switching fusion communication system, as shown in Figure 5, only one of the FSO or RF/THz links is used as a data transmission link, and the other is used as a backup link. The system determines the data transmission path based on the degree of impact of the two links under different weather conditions. To avoid frequent switching between the two links and ensure the maximum utilization of the FSO link, people have proposed a variety of switching algorithm designs. According to the threshold judgment principle, the hard switching mechanism can be divided into single threshold switching, multi-threshold switching, and intelligent threshold switching.

- **Single-threshold switching:** Single-threshold switching is one of the earlier research directions and has made a series of important progress. In 2009, Nadeem et al. conducted a study on an RF/FSO fusion system with single threshold switching based on bit error rate [108]. When the bit error rate of the FSO link is lower than the set threshold, RF is enabled as a backup link. Their research results show that the multi-band switching system can achieve reliability close to the operator level. In 2016, Touati et al. proposed a single threshold switching algorithm based on the received signal-to-noise ratio [109]. When the received signal-to-noise ratio of the FSO link is lower than the set threshold, RF is enabled as a backup link. The effectiveness of this method in overcoming the sensitivity of the FSO link to atmospheric environment changes and aiming errors was verified. However, when the channel quality of the FSO link hovers near the threshold, the single threshold switching scheme may cause frequent switching of the communication link, which will hurt the link performance.
- **Multi-threshold switching:** In 2019, Sharma et al. further proposed a multi-threshold RF/FSO dual-hop transmission system based on the received signal-to-noise ratio [110]. By setting a dual judgment threshold hysteresis interval for the FSO link, the problem of frequent link switching was effectively avoided. In 2021, Shah et al. introduced the maximum ratio merging algorithm into the hybrid link [111]. When the signal-to-noise ratio of the FSO link is lower than the threshold, the maximum ratio merging of the RF and FSO links will be performed to further obtain performance gains. In 2022, Singya et al. studied the theoretical performance of the multi-threshold switching system of THz/FSO [112]. The research results show that the THz/FSO system can better achieve seamless transmission and significantly reduce the average bit error rate of the hybrid system, providing important theoretical support for the subsequent research on THz/FSO fusion communication.
- **Intelligent threshold switching:** The aforementioned switching models generally adopt a passive switching mechanism, that is, the transmitting system needs to wait for feedback information before making corresponding threshold judgments and switching, which inevitably introduces system delays or link interruptions, neural networks (NNs), especially deep learning models, have become powerful tools for addressing the complex challenges of THz and FSO communication systems. In intelligent switching strategies, NNs provide an end-to-end learning framework, bringing intelligence and adaptability to THz/FSO systems. They play a significant role in channel modeling and channel state prediction, facilitating proactive, early switching. In 2016, Nock et al. introduced a Kalman filter to predict the change of received power [113], thereby switching in advance before the link deteriorates. This method effectively prevents frequent switching and significantly reduces the impact on link throughput. In 2017, Guoru et al. first introduced deep learning into the field of channel state prediction [114], showing performance superior to traditional prediction methods. In 2019, Amirabadi et al. verified the effectiveness of deep learning in FSO atmospheric turbulence channels [115]; in the same year, Yejun et al. applied recurrent NNs to the prediction of FSO link received signal strength [116], and made threshold judgments and switching based on the predicted values, realizing a paradigm shift from “switching after failure” to “switching before degradation”. In 2024, Chong et al. deployed a DNND decision model in a THz/FSO system for the first time [107]. They used fog sensor data and channel status as joint inputs and directly output switching decisions through the trained neural network model. In outdoor experimental verification, this method demonstrated extremely high reliability.

#### 4.2.2. Adaptive Soft Switching Strategy

One problem with the hard switching method is the frequent switching between the FSO link and the RF/THz link, which affects the link performance and increases the implementation complexity. In the soft switching scheme, as shown in Figure 5, the FSO and RF/THz links transmit data simultaneously. The hybrid system judges the channel transmission environment based on the channel state information fed back by the receiving end, and then adjusts the transmission ratio of the data in the two links, the system data rate, or the coding rate, and other parameters to avoid switching delays. According to the coding principle, soft switching is mainly divided into hybrid channel coding, hybrid modulation, and adaptive coding.

- **Hybrid channel coding:** In 2009, Hranilovic et al. proposed a hybrid coding method that divides the original data into two parts and then performs short-length Raptor code encoding on each part [117]. This scheme does not require channel information and can adjust the code rate according to the FSO and RF channel conditions, thereby increasing the data transmission rate while reducing the decoding consumption. In 2012, Tang et al. seamlessly maximized the channel throughput by adaptively allocating the amount of data, adjusting the symbol rate, and the encoder rate [118]. In 2023, Amay et al. intelligently adjusted the code rates of the two channels in real time based on artificial intelligence for different channel states [119], maximizing mutual information while enhancing the reliability of the overall link.
- **Hybrid modulation:** In 2009, He et al. first applied joint bit-interleaved coded modulation to hybrid communication systems [120]. By using joint coding and interleaving to better combat the instantaneous impact of deep fading, they achieved seamless integration of RF and FSO channels and proved the excellent performance of hybrid modulation schemes under various weather conditions. In 2015, Tang et al. proposed a hybrid modulation scheme based on P-LDPC code [121]. The scheme maps two bits to the FSO link and one bit to the RF link to obtain three-dimensional dual-link hybrid modulation symbols, and further optimizes the symbol mapping to improve the performance of RF/FSO hybrid communication systems. In 2024, Merrouche et al. proposed adaptive modulation and combination technology [122], which improves the performance by 7 dB compared to hard switching and more than doubles the system service quality.
- **Adaptive coding:** In 2014, Mai et al. proposed a multi-modulation switching technology [123], which significantly outperformed the traditional switching scheme by combining hard switching between two links and adaptive rate soft switching on each link. Following this, in 2017, I.K.Son et al. proposed a dynamic rate allocation technology [124], which dynamically adjusted the dual-link rate through adaptive coding, which could maximize the system reliability and bandwidth utilization. On this basis, in 2019, Qian et al. first applied deep learning channel state prediction and adaptive coding to satellite-to-ground communication [125]. This study presets 9 different coding methods by changing the modulation format (from QPSK to 32 PSK) and adjusting the LDPC code rate, thereby obtaining an error-free threshold adjustment range of nearly 16 dB. According to the channel prediction results, the system can adaptively adjust the coding method to prevent the bit error rate from being too high, significantly improving the channel spectrum utilization while reducing the communication interruption rate.

In recent years, adaptive coding technology based on PCS has shown broad prospects in this field. This technology can provide nearly continuous fine rate adjustment [126,127]. In 2020, Elzanaty et al. designed an asymmetric probability shaping for FSO backhaul links using intensity modulation [128] to counteract the impact of turbulence on the channel, significantly improving the robustness of the link. In 2020, Guiomarr et al. innovatively designed an adaptive coding algorithm based on probability shaping technology [70]. The algorithm predicts the state changes of the FSO channel in real time through the time window averaging algorithm, and adjusts the error-free communication threshold by adjusting the probability shaping parameters, realizing adaptive continuous rate control for the first time. In 2022, they further proposed an RF/FSO adaptive rate complementary system [106]. The system dynamically adjusts the RF link spectrum efficiency when the FSO link rate decreases through probability shaping technology, thereby keeping the total link rate constant, and successfully realizing a highly reliable constant-rate hybrid communication system. In 2025, Xin et al. used photodiode current as channel information feedback [129] and optimized probability distribution in real time based on a genetic algorithm, further demonstrating the excellent performance of adaptive probability shaping in combating atmospheric turbulence. At present, the research on soft switching technology mainly focuses on FSO and RF fusion systems. However, the transmission rate of FSO and THz is similar, which provides greater potential and broader development space for achieving seamless switching.

## 5. Challenges and Future Directions

While significant progress has been made in the integration of THz and FSO communication, several challenges and opportunities remain, summarized in Table 6, majorly in five parts.

- **Challenge 1: Channel Modeling and Attenuation Imperfections**
  Integrated THz/FSO systems face significant signal degradation due to distinct attenuation mechanisms. THz channels are primarily constrained by molecular absorption (e.g., water vapor resonance) and weather-induced attenuation (e.g., rain), which cause high path loss and limit transmission distances to a few kilometers. Conversely, FSO channels suffer from atmospheric turbulence, fog, and pointing errors, leading to random fading and reliability issues. These impairments are exacerbated in dynamic environments like SAGIN, where factors such as ionospheric effects and mobility further complicate channel predictability. Current models often treat THz and FSO channels in isolation, lacking a unified framework to capture their complementary behaviors under varying conditions.
**Future Direction: Development of Unified and Adaptive Channel Models**
  Future research should prioritize the creation of integrated channel models that combine THz and FSO propagation characteristics, incorporating machine learning and deep learning techniques for real-time adaptation. For instance, machine learning algorithms can dynamically predict attenuation patterns based on environmental sensors, enabling proactive compensation. Additionally, leveraging AI for turbulence tracking and absorption mitigation—such as using hybrid neural networks to model spatiotemporal variations—can enhance model accuracy. Standardized datasets from real-world deployments (e.g., SAGIN testbeds) will be crucial for benchmarking and validation, facilitating robust system design for 6G applications.
- **Challenge 2: Hardware Limitations and Transceiver Inefficiencies**
  Practical implementation of THz/FSO systems is hindered by hardware constraints. THz components, including high-power sources and amplifiers, struggle with low efficiency due to parasitic effects (e.g., capacitance losses), restricting output power and leading to limited link distances. FSO systems require precise alignment mechanisms (e.g., ATP), but conventional ATP methods are inadequate under turbulence or mobility. Both technologies face integration challenges, such as mismatched data rates between THz and FSO streams, and lack of compact, cost-effective transceivers for seamless coexistence.
**Future Direction: Advancements in Semiconductor Technologies and Shared Architectures**
  Innovations in semiconductor devices, particularly III-V materials (e.g., GaN, InP), can yield high-gain antennas and efficient power amplifiers for THz bands, extending transmission ranges. For FSO, deep learning-based ATP systems—using convolutional neural networks for beam alignment—can improve robustness. Future work should also explore shared transmitter architectures (e.g., common light sources for THz and FSO) to reduce costs and enhance interoperability. Prototypes focusing on CubeSat-compatible hardware will address SAGIN needs, while photonic integration techniques can unify signal processing chains.
- **Challenge 3: Dynamic Resource Allocation and Switching Strategies**
  Integrated systems rely on adaptive switching (e.g., hard or soft switching) to maintain reliability, but current approaches suffer from latency, frequent unnecessary mode changes, and suboptimal resource utilization. Hard switching causes interruptions due to threshold-based triggers, while soft switching requires complex algorithms for simultaneous data flow, often leading to unstable BER in volatile environments. Moreover, resource allocation lacks intelligence in balancing throughput, spectral efficiency, and energy consumption under constraints like high mobility in SAGIN.
**Future Direction: AI-Driven Adaptive Algorithms and Cross-Layer Optimization**
  Future efforts should emphasize AI-enabled strategies, such as reinforcement learning for real-time switching decisions and resource management. Deep neural networks can predict channel states to enable “switching before degradation,” minimizing delays. Hybrid modulation and coding schemes (e.g., probabilistic shaping combined with orthogonal time frequency space waveforms) can optimize bandwidth utilization. Integration with edge computing will allow dynamic task scheduling, while federated learning frameworks can ensure privacy in distributed networks. Standardization of switching protocols (e.g., via IEEE) will promote interoperability.
- **Challenge 4: Reliability and Resilience in Complex Environments**
  THz/FSO systems must achieve ultra-reliable transmission for critical applications (e.g., deep-space communication or urban 6G networks), but they remain vulnerable to environmental disruptions. FSO links are highly sensitive to fog and turbulence, while THz performance drops in humid conditions. In SAGIN, issues like satellite mobility, space debris, and interference amplify these risks, resulting in unpredictable link outages. Existing systems lack effective mitigation techniques for cross-domain threats, such as simultaneous weather and mobility impacts.
**Future Direction: Intelligent Sensing-Computing-Communication Integration and Debris Detection**
  Research should focus on joint communication and radar sensing frameworks, where THz-based sensing provides environmental feedback (e.g., debris detection with centimeter resolution) to preemptively adjust communication parameters. AI-empowered networks can fuse data from multiple sensors (e.g., fog detectors) to enhance resilience. Additionally, waveform designs like affine frequency-division multiplexing can balance sensing accuracy and data rates. Future directions include developing THz-based debris monitoring systems and robust MAC protocols for SAGIN, leveraging digital twins for simulation-based validation.
- **Challenge 5: Standardization and Practical Deployment Gaps**
  Despite theoretical advances, integrated THz/FSO systems face hurdles in real-world deployment due to the absence of standardization, model architectures, and benchmarking. AI-based solutions often rely on simulated data, limiting applicability. Interoperability issues arise from heterogeneous hardware, and regulatory constraints (e.g., spectrum allocation) hinder scalability.
**Future Direction: Collaborative Standardization and Benchmarking Initiatives**
  Accelerated efforts by bodies like IEEE, ITU, and ETSI are needed to define standards for parameters such as modulation classifications and channel estimation. Creating open-source datasets with real-world turbulence and weather effects will enable reproducible machine learning model training. Future work should also promote hardware-software co-design, ensuring algorithms are optimized for embedded systems in UAVs or satellites. Emphasis on green technologies (e.g., energy-efficient amplifiers) will align with sustainability goals.

## 6. Conclusions

This article provides a comprehensive review of channel transmission models, historical developments, and the current state of THz and FSO systems for high-capacity, long-haul converged transmission. It further explores reliability enhancement solutions for integrated transmission architectures. Through an in-depth analysis of representative advancements, we demonstrate that THz and FSO communications have achieved significant milestones, including Tbps transmission over kilometer-scale distances. Substantial progress has also been evidenced in hybrid RF/FSO system design and reliability-driven hard/soft switching technologies. Currently, research focus is shifting toward seamless THz/FSO systems, where initial breakthroughs have been validated. Our analysis reveals that integrated THz/FSO communications hold transformative potential for 6G networks and SAGIN, offering high capacity and reliability by leveraging complementary strengths. However, key challenges—spanning channel modeling, hardware limitations, resource allocation, resilience, and standardization—must be addressed to realize this potential. Critical findings indicate that AI and machine learning technologies are pivotal for dynamic adaptation, while advancements in semiconductors and shared architectures can overcome hardware bottlenecks. Future success hinges on interdisciplinary collaboration, emphasizing unified models, intelligent switching mechanisms, and practical deployments. By prioritizing these directions, integrated THz/FSO systems can evolve into robust, scalable infrastructures capable of meeting the demands of next-generation networks.

## Figures and Tables

**Table 1:** Intensity range of rain.

Intensity	Range of R (mm/h)
Very light	*R* < 1
Light	1 ≤ *R* < 2
Moderate	2 ≤ *R* < 5
Heavy	5 ≤ *R* < 10
Very heavy	10 ≤ *R* < 20
Extreme	*R* ≥ 20

**Table 2:** Performance comparison of different communication systems.

	FSO	RF	THz	RF/FSO	THz/FSO
Spectrum resources	High	Low	High	Low/High	High/High
Transmission rate	High	Low	High	Low/High	High/High
Atmospheric turbulence impact	High	Low	Low	Low	Low
Rain impact	Medium	Low	High	Low	Medium
Fog impact	High	Low	Low	Low	Low
Perception capability	Low	High	High	High	High
Confidentiality	High	Low	Relatively high	Relatively low	Relatively high
Reliability	Low	High	Relatively high	High	High

**Table 3:** Representative progress in THz and FSO high-capacity communication research.

Wavelength	Techniques	Capacity	Distance	Reference
THz	Deep neural network equalization, high-order modulation, MIMO and link optimization	107.52 Gbps	36 m	[59]
	High-order QAM combined with PCS	109 Gbps	1.1 m	[60]
	Kramers–Kronig receiver and optical injection locking technique	120 Gbps	1.2 m	[61]
	Optimizing Links with Cassegrai Antennas	220 Gbps	214 m	[62]
	Channel equalization using likelihood-aware vector quantization variational autoencoder	366.4 Gbps	6.5 m	[63]
	Airy beam, multi-stream low interference design	400 Gbps	0.7 m	[64]
	High order QAM, multi-band	938 Gbps	0.12 m	[65]
	Frequency, polarization and space division multiplexing	1.034 Tbps	100 m	[66]
	80-channel WDM	6.4 Tbps	54 m	[67]
FSO	QPSK and 3-channel WDM	120 Gbps	1 km	[68]
	Link optimization using dual polarization and linear polarization combined with OCDMA	240 Gbps	1.41 km	[69]
	Using the time domain memory and channel estimation algorithm of the FSO link, combined with PCS	400 Gbps	55 m	[70]
	Link optimization using cylindrical vector beams, 8-channel WDM	640 Gbps	3 km	[71]
	PCS dynamic modulation, ATP active alignment, high-order modulation	1 Tbps	3 m	[72]
	High-order QAM and adaptive optics mitigate the effects of atmospheric turbulence	1 Tbps	53 km	[73]
	Link optimization and 102-channel WDM using micro-cavity soliton frequency comb	1.02 Tbps	1 km	[74]
	Delta–Sigma modulation 1024QAM combined with 96-channel WDM	7.68 Tbps	0.1 km	[75]
	9-hole transmit and single-hole receive link optimization, PCS, 35-channel WDM	14 Tbps	220 m	[76]

**Table 6:** Challenges and future research directions.

Challenges	Key Issues	Future Research Directions
Channel Modeling & Attenuation	Lack of unified model; THz molecular absorption; FSO turbulence	Develop ML-based unified channel models for dynamic adaptation
Hardware Limitations	THz low power efficiency; FSO alignment precision; transceiver integration	Advance semiconductor tech (e.g., GaN/InP) and shared architectures
Resource Allocation & Optimization	Seamless switching challenges; resource utilization	AI-driven adaptive algorithms (e.g., reinforcement learning)
System Reliability & Resilience	Vulnerability to weather/mobility; link outages in SAGIN	Integrate sensing and AI for proactive resilience
Standardization & Deployment	Lack of standards; interoperability gaps	Collaborative standardization and real-world testbeds

## Data Availability

The data presented in this study is available on request from the corresponding author. The data is not publicly available due to privacy restrictions.
